# Voluntary Health Insurance expenditure in low- and middle-income countries: Exploring trends during 1995–2012 and policy implications for progress towards universal health coverage

**DOI:** 10.1186/s12939-016-0353-5

**Published:** 2016-04-18

**Authors:** Luisa M. Pettigrew, Inke Mathauer

**Affiliations:** Department of Health Services Research and Policy, London School of Hygiene and Tropical Medicine, 15-17 Tavistock Place, London, WC1H 9SH UK; Department of Health Systems Governance and Financing, World Health Organization, Avenue Appia, Geneva, Switzerland

**Keywords:** Private health insurance, Voluntary health insurance, Universal health coverage, Low- and middle-income countries, Health expenditure

## Abstract

**Background:**

Most low- and middle-income countries (LMIC) rely significantly on private health expenditure in the form of out-of-pocket payments (OOP) and voluntary health insurance (VHI). This paper assesses VHI expenditure trends in LMIC and explores possible explanations. This illuminates challenges deriving from changes in VHI expenditure as countries aim to progress equitably towards universal health coverage (UHC).

**Methods:**

Health expenditure data was retrieved from the WHO Global Health Expenditure Database to calculate VHI, OOP and general government health (GGHE) expenditure as a share of total health expenditure (THE) for the period of 1995–2012. A literature analysis offered potential reasons for trends in countries and regions.

**Results:**

In 2012, VHI as a percentage of THE (abbreviated as VHI%) was below 1 % in 49 out of 138 LMIC. Twenty-seven countries had no or more than five years of data missing. VHI% ranged from 1 to 5 % in 39 LMIC and was above 5 % in 23 LMIC. There is an upwards average trend in VHI% across all regions. However, increases in VHI% cannot be consistently linked with OOP falling or being redirected into private prepayment. There are various countries which exhibit rising VHI alongside a rise in OOP and fall in GGHE, which is a less desirable path in order to equitably progress towards UHC.

**Discussion and Conclusion:**

Reasons for the VHI expenditure trends across LMIC include: external influences; government policies on the role of VHI and its regulation; and willingness and ability of the population to enrol in VHI schemes. Many countries have paid insufficient attention to the potentially risky role of VHI for equitable progress towards UHC. Expanding VHI markets bear the risk of increasing fragmentation and inequities. To avoid this, health financing strategies need to be clear regarding the role given to VHI on the path towards UHC.

**Electronic supplementary material:**

The online version of this article (doi:10.1186/s12939-016-0353-5) contains supplementary material, which is available to authorized users.

## Background

Countries are increasingly determined to move towards universal health coverage (UHC). This involves creating financing mechanisms to ensure “that all people can use the health services they need, of sufficient quality to be effective, while also ensuring that the use of these services does not expose the user to financial hardship” [1]. This progress towards UHC should be equitable in that lower income and other vulnerable or disadvantaged population groups should equitably benefit from progress rather than being left out until later [2]. Yet many low- and middle-income countries (LMIC) are still far from reaching UHC, as they rely significantly on private health expenditure (PvtHE) as a share of total health expenditure (THE). Most of PvtHE is made up of regressive out-of-pocket payments (OOP). Another part of PvtHE is private health insurance, now more frequently referred to as voluntary health insurance (VHI), which is also usually a more regressive form of prepayment than tax revenue or social security funds (referred to here as public health insurance) and offers a more limited form of risk sharing. Overall, PvtHE is thus a more inequitable form of health financing, through which it is more difficult to progress towards UHC in an equitable way.

This paper focuses on VHI in LMIC. Increases in VHI expenditure as a mechanism to finance health systems have implications on countries’ efforts to move towards UHC. We do not argue that there is a threshold of VHI share that is harmful for UHC progress, but global evidence suggests that VHI is not a suitable mechanism to move towards UHC in an equitable way given its regressive nature and other reasons outlined further below [1]. It is therefore important to be aware of trends in VHI expenditure and to respond to potential challenges deriving from changes in VHI expenditure and the role VHI plays.

For countries of the Organisation for Economic Co-operation and Development (OECD) and the European Union various reviews of VHI expenditure trends, often previously referred to private health insurance (PHI), as well as country specific analyses are available [3–7]. Sekhri and Savedoff (2005) provide a global overview and descriptive analysis of the share of PHI from THE in 2001. They indicate that in 2001 PHI expenditure was above 5 % of THE in 39 countries worldwide, 25 (64 %) of these being LMIC, pointing out how widespread PHI had become across the globe [8]. Drechsler and Jütting (2005) provided an analysis of PHI as a share of THE from 1997 to 2001 based on National Health Accounts (NHA) [8, 9] which included LMIC, and gave an update for the period 1998-2002. They observed a rising, although so far overall small PHI shares of THE in LMIC [10, 11]. To note, globally, there is no relationship (R2 = 0.01) between GDP per capita and VHI% [12], which was also earlier observed by Drechsler and Jütting [10].

However there are no more recent publications presenting and exploring overall trends in VHI expenditure globally in LMIC with NHA data after 2002. The significance and role of VHI may have changed during this period as many LMIC have begun to place more attention on their health financing system. The purpose of this paper is therefore to provide a comprehensive overview of VHI expenditure trends in LMIC and to explore potential reasons for these trends throughout the whole period of available NHA data since 1995 and with an additional 10 years of data since the previous studies. This NHA data is made available through the World Health Organization’s (WHO) Global Health Expenditure Database (GHED) [13]. As such, this paper also serves as a data repository on VHI expenditure as a basis for further country analysis and future research.

Voluntary health insurance (VHI) schemes are based voluntary prepayment of premiums with the mode of participation being upon the discretion of an individual or a firm or group. Benefits are agreed between the beneficiary and insurer. This is in contrast to compulsory schemes where membership and payment of contributions are made compulsory by the government (by law) for the population as a whole or large sections of the community. VHI appears in many forms: there are large commercial schemes as well as smaller non-profit ones. They include employer-based insurance contracts, and community-based health insurance (CBHI) [14].

There are different categorizations of the role of VHI in relation to public health insurance [5, 6]. The OECD categorization of VHI functions distinguishes between the following: Primary; complementary; supplementary; or duplicative [5] which are further explained in Table 1. In practice, several roles for VHI for different population groups may co-exist in a country. It is often difficult to distinguish primary from supplementary VHI coverage when there is only a vaguely defined package of services, in particular if they are of poor quality, or actually not available in practice.

**Table 1:** Classification of VHI roles

VHI role	Country examples
Primary	
Principal: Represents the only available access to health insurance.	In the United States of America, VHI has been the principal source of coverage before 2014 [78].
Substitutive: Substitutes for cover which would otherwise be available from the public health insurance system, but the individual has voluntarily opted out of this or is not entitled to it. In the case of opting out, people do not pay public health insurance contributions.	In Germany and Chile, opt out options from the public health insurance system exist in order to be covered by VHI [3, 47]. Such substitutive VHI often includes additional services and is thus also supplementary in nature.
Complementary	
Complements coverage of publicly insured services or services within principal/substitute health insurance, by covering all or part of the residual costs (e.g. co-payments).	France’s complementary VHI primarily serves to reimburse copayments required in the public health insurance system [79].
	Community based health insurance schemes in Sub-Saharan Africa usually cover user fees, although they may also represent a form of principal voluntary health insurance.
Supplementary	
Covers additional health services not covered by the public scheme; depending on the country this may include for example elective care, long-term care, dental care, pharmaceuticals, rehabilitation, alternative or complementary medicine, superior hotel and amenity hospital services.	In Germany supplementary VHI exists for additional services (e.g. dental care, private hospital room). Similarly supplementary VHI is found in the Russian Federation, Latvia and Hungary [80].
Duplicative	
Covers health services already covered under public health insurance, but with access to other, additional providers or levels of service, e.g. private health facilities. However unlike substitutive insurance it does not exempt enrollees from contributing to public health insurance.	In the United Kingdom duplicative VHI exists as an additional alternative to the public system.
	Similarly in Nicaragua, wealthy people may purchase VHI that provides access to the private sector and coverage of additional services that are not included in the mandatory public scheme. Yet, they continue paying public health insurance contributions [81].

Source for classification of VHI roles [5]

The extent of advantages and disadvantages of VHI depends on its specific type. Each of the four types outlined above has potential positive (foremost reducing OOP expenditure) and negative implications in progressing towards UHC, through specific effects on access, equity and efficiency from a health system perspective. When prepayment levels through compulsory sources are minimal, VHI as a form of prepayment and limited pooling could be preferable to OOP expenditure, as it may expand financial protection and access to additional services [1]. VHI has also been argued to potentially bring more money into the health system and to cross-subsidise at provider level as well as to enhance uptake of new technologies [5, 6, 9, 15]. However, VHI can suffer from a number of market failures due to adverse/risk selection, leading to a spiral of increasing premiums, further enhancing adverse selection, thus potentially making VHI unaffordable and creating market instability. VHI can significantly contribute to fragmentation and unbalanced risk pools in public health insurance with a larger proportion of sicker patients. It builds up barriers to redistribute funding in favour of the poor or sick and thus increases inequity in access if not well regulated. VHI can also lead to increased use of unnecessary health care due to moral hazard and supplier-induced demand, this may be a particular issue for supplementary and complementary VHI.

Most importantly due to group interests and associated resistance, it may be politically more difficult to introduce or expand compulsory prepayments to finance coverage extension for less affluent population groups when a VHI market is already in place. This is can be seen in particular where primary VHI coverage exists, even if it only covers a small percentage of the population or contributes a modest amount to the share of THE.

The next section describes the methodology for this study. The results presents trends in VHI expenditure in LMIC and identifies factors that may explain these. A discussion follows which brings together three emergent themes of factors from a global perspective which may influence the development and role given to VHI. Finally conclusions, policy lessons and implications in relation to VHI and equitable progress towards UHC are offered.

## Methods

This study is based on data from the WHO’s GHED downloaded in December 2014. This provided data for the years 1995–2012. Looking at the whole period for which data is available helps to identify broader trends and adds an additional 10 years of data to previous publications. The GHED covers all member states of the World Health Organization, currently 194, and provides a set of health expenditure data [13]. Data is added and updated on a yearly basis by the WHO Health Accounts team. Each country makes data available in a standardized format by following the Health Accounts methodology, considered as the international standard [14, 16]. The Health Accounts methodology outlines basic accounting criteria and guidance on timeliness, data completeness, consistency and accuracy as well as data validation and triangulation. This guidance aims to increase the reliability of both public as well as private expenditure data collection.

However of particular concern is the data quality of VHI expenditure data. The OECD’s Systems of Health Accounts and WHO’s Guide to Producing Health Accounts note that collecting data on VHI expenditure is one of the most challenging out of all the health expenditure types. As the NHA guidebook states: “Incomplete sources and estimation methods of private expenditure on health are among the major limitations for international comparison” [16]. One specific challenge is that health insurance may be part of other types of insurance (e.g. life insurance) or health insurance may cover benefits beyond healthcare (e.g. income benefits) which may be hard to disaggregate. Moreover, a fast turnover of VHI organisations and limited or immature market regulation can result in poor quality VHI data being provided. These factors alongside others around the timing of data collection and movement of funds between the insurer, policy holder and healthcare provider, as well as the existence of reserve and surplus funds and administrative costs, create potential limitations to the accuracy of data [14, 16].

It also should be noted that the underlying terminology and definitions for the NHA data currently available in GHED is based on the System of Health Accounts 1.0 [17] which uses the term private health insurance (PHI). However, the newer System of Health Accounts (SHA) 2011 edition has modified the classification and terminology in order to be more precise and employs the term voluntary health insurance [14]. In line with the SHA 2011 terminology, this paper uses the term voluntary health insurance to refer to private health insurance, although data is based on the previous definitions.

The GHED includes estimates on GGHE (general government health expenditure) and PvtHE (private health expenditure) as percentages of THE (total health expenditure) and as expenditure in national currency units. It also provides OOP and VHI expenditure as a percentage of PvtHE. Importantly the VHI data available does not identify what role VHI is playing as outlined in Table 1. Only further country context information and literature analysis allows exploration of which VHI role prevails.

VHI expenditure as a share of THE was calculated based on the share of PvtHE in THE multiplied by the share of VHI expenditure in PvtHE. VHI expenditure as a percentage of THE is abbreviated as “VHI%” as are “OOP%” and “GGHE%” when respectively referring to OOP and GGHE as a percentage of THE.

To explore VHI expenditure trends countries were grouped by the WHO Regions as follows; African Region (AFRO) Region of the Americas (AMRO), Eastern Mediterranean Region (EMRO), European Region (EURO), South East Asian Region (SEARO) and Western Pacific Region (WPRO). Whilst there are differences between countries, this grouping is useful in that LMICs in these regions show, on the whole, geographical and socio-economic similarities. The 2012 World Bank classification of country income levels was used to categorize countries into low, lower-middle, upper-middle and high income. Countries which moved into the high income classification between 2010 and 2012 were still included in the LMIC country trend analysis. Otherwise their exclusion could have missed relevant information on VHI trends as during the majority of the period in question (1995–2012) they were considered as LMIC.

Countries with no data or more than five years of data missing on VHI expenditure during 1995–2012 were excluded from the calculation of the VHI% average by WHO region. For country trend analysis within each regional section below countries having had a VHI% ≥ 1 % at least once during the last 5 years of the observation period (i.e. 2008–2012) were included.

Grouped by WHO Regions the following individual country data analyses are presented:

1. VHI% trends between 1995–2012
2. Summary tables in the Addendum (Additional file 2) with:
  - the direction in change (rise or fall) of VHI, OOP and GGHE as a share of THE (VHI%, OOP%, GGHE%) between 1995 and 2012
  - OOP% of THE in 2012, grouped into 3 categories (below 20 %, between 20–40 % and above 40 %)

Changes in per capita expenditure in purchasing power parity dollars in VHI, OOP and GGHE were also analysed. As was THE as a percentage of GDP. These have not been presented in this paper but have helped informed the results and analysis. In addition to assessing VHI expenditure trends, potential reasons for the trends in VHI expenditure were explored and interpreted based on analysis of the literature. A literature search of English publications was undertaken in PubMed and Web of Knowledge starting from 1990 to June 2015. Grey literature was searched for in Google, for which the first 7 pages (70 results) were screened for relevant material. Titles identified through the search process were reviewed, and if found to be relevant the abstract or executive summary was read. If this suggested that the publication could provide a reason for the trend in VHI expenditure the full publication was assessed. Additional papers were identified through the reference list of literature identified, as well as through the authors’ knowledge of relevant publications. Potential explanations for trends in VHI expenditure were recorded and then analysed to identify emerging themes. Health sector and health financing strategy documents available on the IHP+ Platform were screened for whether they mentioned or defined the role of VHI [18].

## Results

### Number of countries with VHI% data

**Fig. 1:**
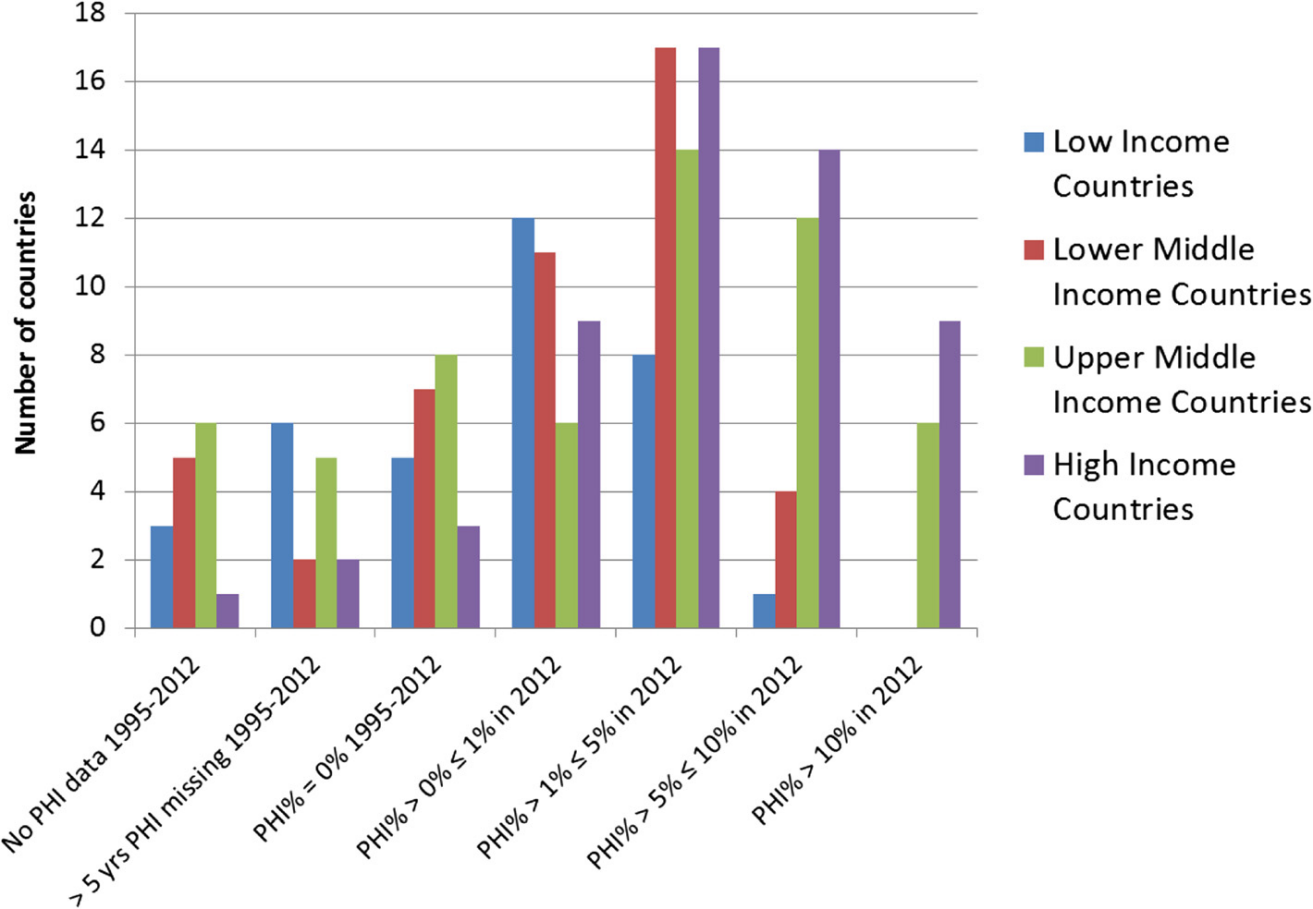
Overview of VHI in 2012 by country income classification

**Fig. 2:**
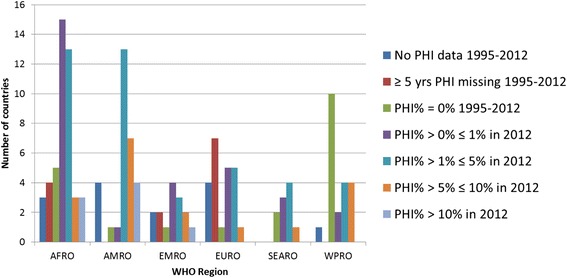
Overview of VHI% in 2012 in LMIC + 7 recent HIC by WHO Region

The GHED provided information on 193 countries, when data was downloaded. Out of these 55 countries were classified as high-income countries (HIC) and 138 as LMIC in 2012. Figure 1 provides a summary of VHI data availability by country income group. Twenty-seven LMIC (20 %) had no data or more than five years of missing VHI expenditure data; these were excluded from further analysis (listed in Additional file 1). This left 111 LMIC with available data. Seven HIC countries (Antigua and Barbuda, Chile, Latvia, Lithuania, the Russian Federation, Uruguay and Saints Kitts and Nevis) were included in the regional analysis and in the literature search as they had been LMIC for the majority of the 1995–2012 period (Fig. 2). From this total of 118 countries, 20 countries reported zero VHI% throughout the period, and 30 countries reported a VHI% of below 1 % in 2012. Countries with a VHI% of below 1 % in 2012 were excluded from the individual country trend analysis and literature search, unless the VHI% had been above 1 % at some point during the period of 2008–2012, which was the case for five AFRO countries and one WPRO country. In total, 74 countries met the final inclusion criteria for individual country trend analysis.

### VHI% trends by country income classification and by WHO region

A total of 46 countries had VHI% > 5 % in 2012, of these 23 were LMIC and three countries had recently become HIC (Fig. 1). Worldwide, 56 countries had VHI% between 1 % and 5 %, 39 of these were LMIC and three had recently become HIC.

Figure 2 provides an overview of VHI% in LMIC in the six WHO regions in 2012. AFRO, AMRO and EURO have the greatest number of countries with missing data. WPRO is the region with highest proportion of countries reporting zero expenditure on VHI. AMRO has the highest proportion of countries with VHI% > 5 % compared to other regions. EURO, SEARO and WPRO have no countries with VHI% > 10 %.

**Fig. 3:**
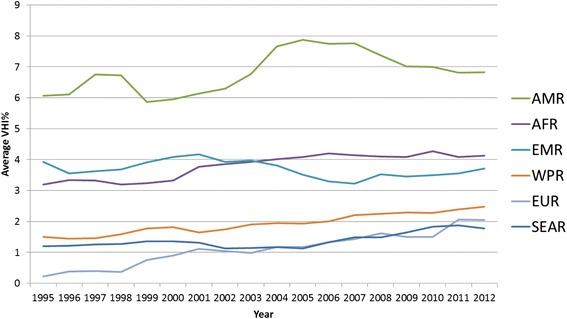
Average VHI% trends in LMIC + 7 recent HIC by WHO Region

Figure 3 shows the VHI% averages of LMIC by WHO region during 1995–2012. With the exception of EMRO countries, since 1995 the average VHI% in all regions has increased slightly. The highest average VHI% is found in AMRO.

### Country VHI% trends in LMIC by WHO region

#### African Region (AFRO)

**Fig. 4:**
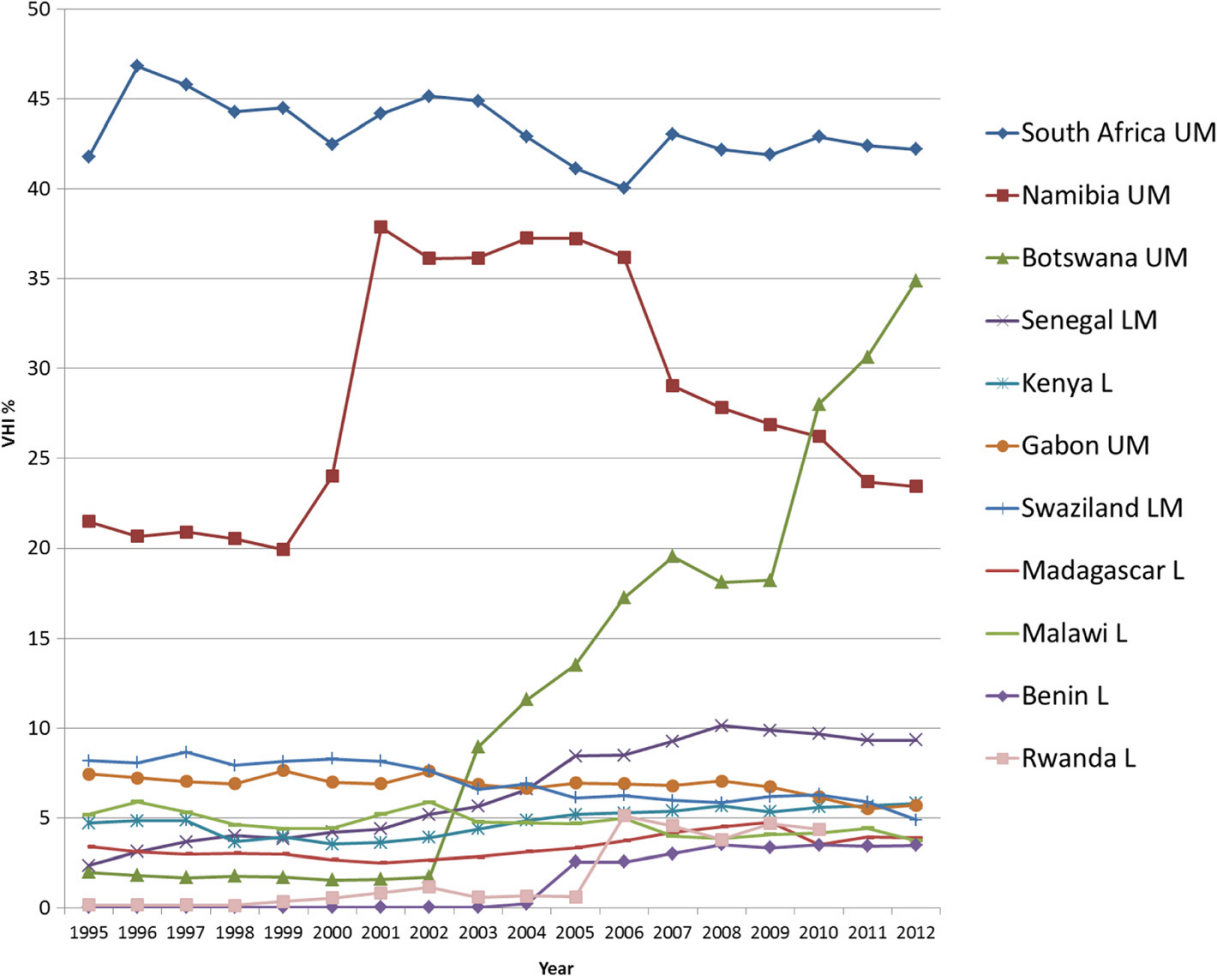
AFRO Individual country trends of VHI%, 1995–2012, VHI% >3 % in 2012

**Fig. 5:**
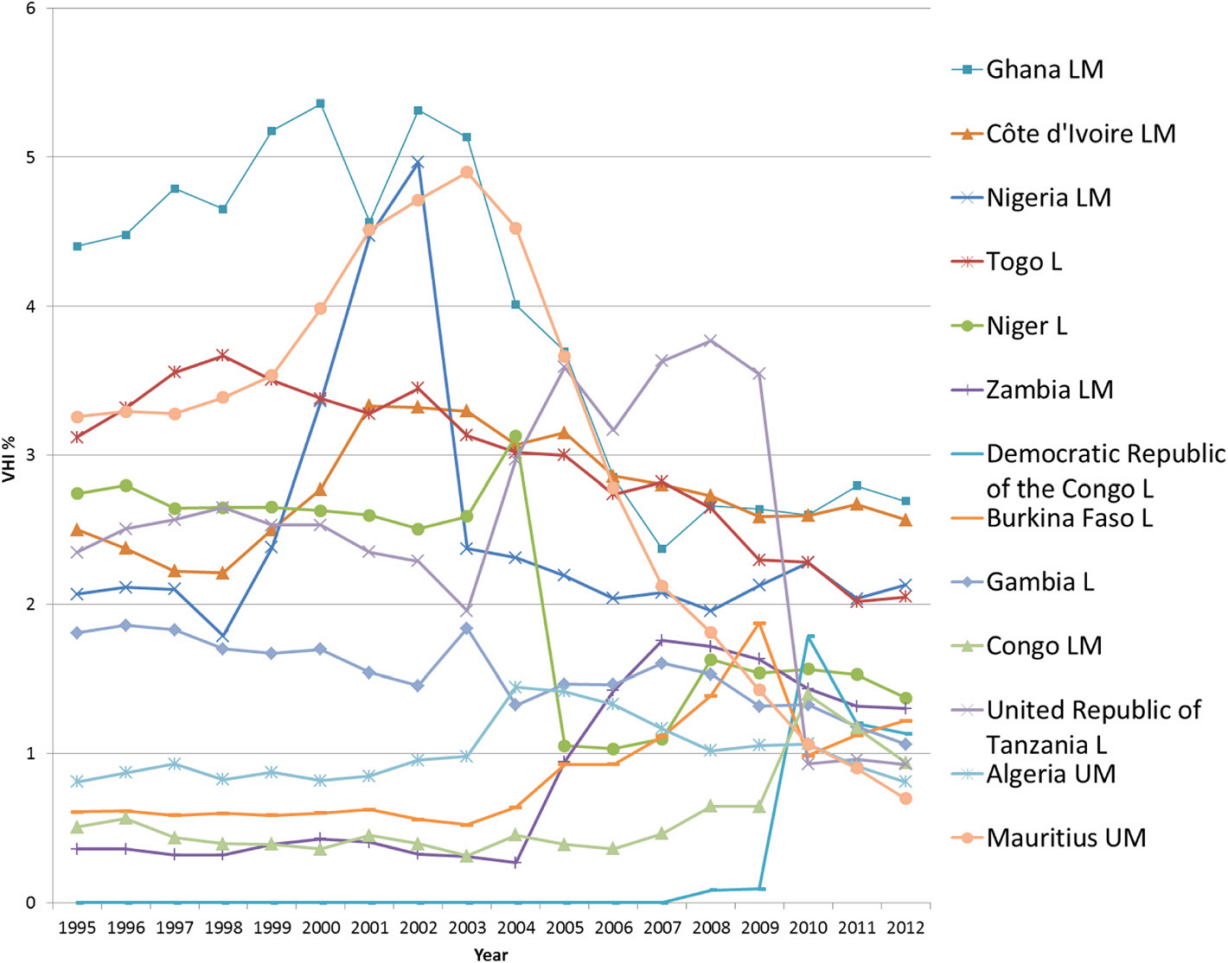
AFRO Individual country trends of VHI%, 1995–2012, VHI% ≤ 3 % in 2012

Out of the 46 AFRO LMIC, VHI expenditure data is recorded for 39 countries. The regional average VHI% for AFRO appears fairly stable between 3.2 %–4.2 % with a slight upward increase (see Fig. 3). Fifteen countries recorded a VHI% of less than 1 % over the past five years in 2012, and five countries reported zero VHI% throughout 1995–2012. There is thus some considerable variance across AFRO countries, as revealed in Figs. 4 and 5, which present individual country trends of VHI% for 24 countries with a VHI% above 1 % between 2008-2012. The data has been split into two graphs to enable reading with Figure 4 showing countries with VHI% <3 % in 2012 and Figure 5 those >3 % in 2012.

South Africa, Namibia and Botswana have a notably much higher VHI% in comparison to other countries in the region, with a significant increase in Botswana after 2002. Overall, though, only six countries have a VHI% above 5 % in 2012. On looking more closely Senegal, Benin and Rwanda respectively saw relatively sharp rises in VHI% during the period in question.

For each country it was assessed whether there was an overall rise or fall in OOP% and VHI% set against a rise or fall in GGHE% (see Additional file 2). It shows that the GGHE% increases in 20 out of 24 countries. In six of these countries, both OOP% and VHI% go down, while in the other 13 countries, VHI% increases, whilst OOP% decreases. Ghana is the only country with rising GGHE as well as an OOP% increase, while VHI% declines. However, in four countries (Tanzania, Mauritius, Namibia and Kenya) GGHE% decreases, and notably the VHI% increases in the latter two of these countries with OOP% also going up. This scenario with a fall in GGHE%, rise in VHI% and rise in OOP% is undesirable with respect to equitably moving towards UHC.

Community based health insurance (CBHI) usually covers user fees and thus primarily serves as complementary insurance, but also may provide additional services in a supplementary role. As such, it has increasingly been perceived as a possible avenue through which to improve healthcare coverage in LMIC in Sub-Saharan Africa. The uptake in the number of CBHI schemes [19] might be one reason for high percentage increases in per capita VHI spending massively outstripping the associated relative percentage increase in THE per capita expenditure between 1995 and 2012, seen in Benin, Burkina Faso and Senegal. Rwanda also showed a substantial rise in VHI% and VHI expenditure per capita, which can be explained by the government's decision in 2005 to nationally roll out the CBHI schemes (Mutuelles de Sante) [20, 21]. These have been counted as VHI even though enrolment has been mandatory in practice and therefore their classification will change under the new system of health accounts. Despite heavy promotion by some development partners and some ministries, rates of CBHI enrolment remain limited in most of the AFRO area [22]. Therefore ultimately, increases in VHI expenditure via CBHI are also limited. Thus, assumingly, some other part of the VHI expenditure increase may be explained by commercial VHI, also in a complementary and supplementary role targeted at wealthier segments of the population, although this remains affordable to very few.

The much higher levels of VHI% preceding 1995 in South Africa and Namibia were due to the long existence of VHI that served the more affluent populations as primary (principal) coverage [11]. A similar development occurred in Botswana, where a rising middle class could afford VHI [23]. Notably, VHI% is much higher than the percentage of the population covered by VHI. The South African legacy of the apartheid period left a very inequitable and fragmented health financing system. VHI (called “medical aid schemes”) had been set up and tailored to the needs of wealthy white urban populations [24]. However, increasing unaffordability of VHI due to high premiums led to a decline in VHI population coverage [25, 26]. There was also a fall in the number of VHI funds [27]. Following the African National Congress coming into power in 1994 there has been progress to address these inequities, reflected in the falling OOP% and rising GGHE% [28], which marginally reduced VHI% in the post-apartheid period. Yet, the introduction of market-oriented policies in 1996, which intended to stimulate the economy were a barrier to quick growth in public spending [29, 30]. It should also be noted that although Zimbabwe has been omitted in this study due to missing data, it also had a substantial percentage of THE spent on VHI previously recorded (18 % in 2001), as reported by Drechlser and Jütting [11].

In summary, in AFRO, there is no consistent trend regarding the VHI expenditure changes: On the one hand, some Southern African countries (South Africa, Namibia, Swaziland) with primary (principal) VHI, which had historically relatively higher levels, show decreasing VHI%, largely due to increased GGHE. Botswana and Senegal show exceptionally large increases of more than 30 and 5 percentage points respectively in VHI%. On the other hand, for countries with VHI% below 8 %, there are both upward and downward developments within the range of 2 % percentage points for most countries. In countries with an overall downward trend, increasing VHI expenditure per capita due to a growing increasing middle class demanding better (supplementary and complementary) VHI coverage as well as the promotion of CBHI schemes may thus be offset by increased government commitment for higher GGHE, which we argue is a more desirable direction on the path to UHC [31].

#### American Region (AMRO)

**Fig. 6:**
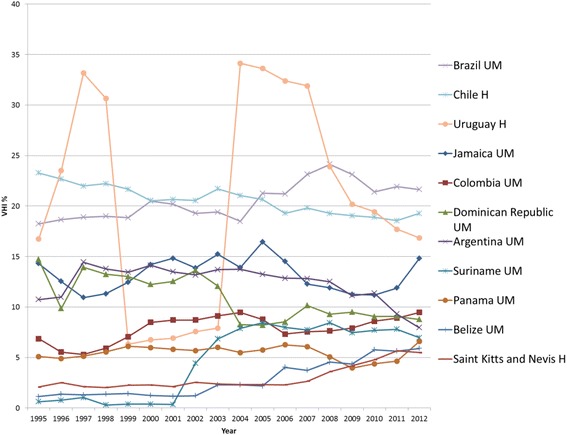
AMRO Individual country trends of VHI%, 1995–2012, VHI% > 5 % in 2012

**Fig. 7:**
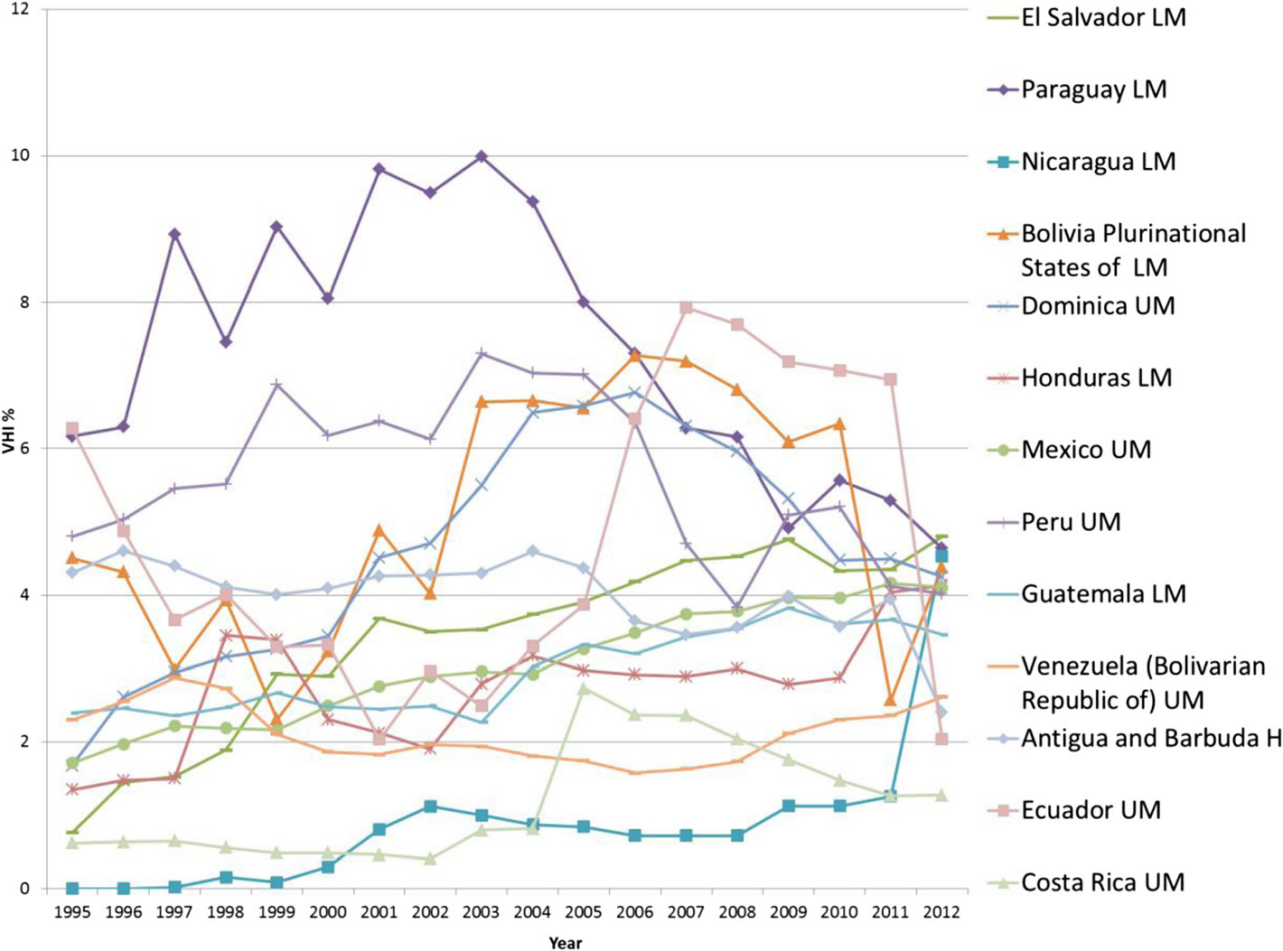
AMRO Individual country trends of VHI%, 1995–2012, VHI% ≤ 5 % in 2012

Out of a total of 30 LMIC in AMRO, 26 have VHI data. Figures 6 and 7  present the individual country trends in VHI% from 1995–2012 for the 24 AMRO countries with VHI% above 1 % in any year between 2008 and 2012. Included are also Chile, Uruguay, Antigua and Barbuda, and Saint Kitts and Nevis, which recently became HIC. Together with AFRO, AMRO has a much wider variation in VHI% compared to other regions. It also has the greatest proportion of countries with VHI% >5 %, and so AMRO has had the highest average VHI% throughout the 18 year period (see Fig. 3). Although none of the AMRO countries maintain as high a VHI% as South Africa and Namibia, five AMRO countries maintained a VHI% of >10 % during all of the 18 year period, and only in the Dominican Republic did the VHI% of 15 % in 1995 decline and reach between 8–10 % from 2004 onwards. Likewise, Argentina shows a declining trend since 2000. There are also several countries in AMRO with relatively sharp variations in VHI expenditure data. In the absence of plausible reasons to explain such steep variations, questions around the data itself arise, in particular in Uruguay where there is a significant dip and then return to the previous trend in the early 2000s. The data has been split into two graphs to enable reading with Figure 6 showing countries with VHI% <5 % in 2012 and Figure 7 those >5 % in 2012.

In seven out of 24 countries included in the analysis, GGHE% decreased, and in most of these, both VHI% and OOP% increased, which is a less desirable health expenditure development in moving towards UHC equitably. On the other hand, in 17 countries, GGHE% increased, with just under half of these also showing an increase in VHI%, while OOP% decreased (see Additional file 2).

For the 18 countries with a VHI% below 10 % in 2012, the overall trend has been for the VHI% to rise, at least during the first half of the period studied. In Suriname, VHI exists in several roles: public health insurance is primarily for civil servants, with voluntary membership for others and the private formal sector is primarily covered via employer based health insurance or other VHI [32]. This covered about 17 % of the population in 2002 [33] and may have contributed to the rises noted in VHI%. In Colombia, the health sector underwent reform in 1993 guided by international financial institutions that contributed to a slight growth of VHI [34]. In the Dominican Republic it was reported that deficiencies in the public tax financed system led to the initial growth of the VHI sector, but in 2001 new laws created a social security system with public health insurance that led to greater GGHE and caused VHI% in turn to fall gradually over the 2000s [35]. In Mexico, the opt-out option from the public health insurance scheme for private sector formal employees and option to take primary (substitutive) VHI instead since 1995 only led to a minor rise in VHI%. This limited growth has been attributed to the lack of regulation to support the private sector's growth in a secure financial and fiscal environment [36]. High VHI premiums constitute an important financial barrier for the majority of the Mexican population. Moreover, the national public health insurance programs have been reported to provide a relatively comprehensive package [37].

In the group of countries with VHI% >10 % in 1995, various of these, e.g. Chile, Argentina, Uruguay and Brazil, have been influenced by market focused reforms which were encouraged by multilateral lending agencies prior to 1995 [38]. Income growth among the wealthier population was also a reason for a rise in the VHI%. In Brazil, for example, 25 % of the population have VHI coverage, largely in its supplementary role [39, 40]. Likewise, in Chile, where opting out of public health insurance was previously allowed, 25 % of the population chose primary (substitutive) VHI coverage by 1997, resulting in a significant rise in VHI%. Of note the VHI schemes also manage the 7 % public health insurance contribution, so only the contribution above the 7 % is VHI in nature and includes supplementary coverage, even though the current GHED data does not disaggregate this and reports all as VHI.

Despite coalitions of health professionals, academics, unions and local communities coming together to resist reforms [38, 41] and with limited regulatory frameworks in place described as a “legal vacuum” [36], the VHI market grew substantially in the 1980s–90s. Health Maintenance Organisations (HMO), often from overseas, emerged, targeting the rising incomes and desires of the wealthier middle-classes [42]. Yet, rising VHI premiums resulted in reduced enrolment rates as found in Argentina [43] and in fact contributed to a decline in VHI%. Importantly, increased investment in GGHE to provide coverage to poor and vulnerable groups in Uruguay, Brazil and Chile coupled with firmer regulation [44, 45] have contributed to a decline in VHI% in recent years. For example in Chile, the introduction of AUGE (Acceso Universal con Garantias Explicitas - Universal Access with Explicit Guarantees) made public health insurance more appealing, which resulted in VHI membership substantially falling and remaining stable over more recent years [46, 47].

In summary, many Latin American countries reached high VHI% levels, largely due to private sector policies in the past as well as due to increasing demands of the population. As a number of countries were strongly committed to expanding GGHE and undertaking equity focused reform in efforts to move towards UHC since the mid-2000s, VHI% has gone down in many settings, especially in those countries which had VHI% levels above 10 % in the 1990s.

#### Eastern Mediterranean Region (EMRO)

**Fig. 8:**
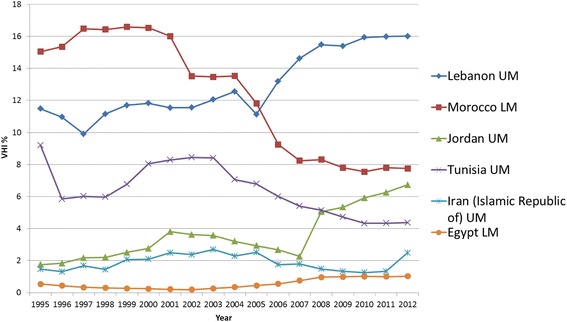
EMRO Individual country trends of VHI%, 1995–2012

The average VHI% is around 3–4 % in the 15 EMRO countries (Fig. 3) that provided VHI expenditure data. There were six countries with a VHI > 1 % in 2012. As Fig. 8 presents, three of these countries (Lebanon, Morocco, Tunisia) had a VHI% above 5 % for the majority of the period in question, and Jordan’s VHI% rose above 5 % in 2008. Morocco has had a VHI% which is noticeably the highest for all lower-middle income countries included in this study. However Morocco and Tunisia show downward trends in VHI%, whereas a strong upward trend is noted for Jordan and Lebanon in the latter 2000s. Iran’s and Egypt’s supplementary/complementary VHI markets are still relatively small at less than 2.5 % of THE.

In four out of six countries (Jordan, Morocco, Lebanon, Tunisia) GGHE% increases. In the remaining two countries (Egypt and Iran), GGHE% decreases with slightly increasing VHI%. OOP% decreases in Iran however it increases in Egypt, this being a less desirable trend with respect to equitably moving towards UHC (see Additional file 2).

In Jordan limited public health insurance and economic policies that promoted trade liberalization and privatization have been offered as explanatory factors for Jordan's steady rise in VHI% [48], where the VHI sector comprises of private insurance companies, self-insured firms, and third party administrators principally for the upper-middle professional classes [49]. In addition coordination between the Ministry of Industry and Trade, which is responsible for VHI regulation, and the Ministry of Health has been reported to be limited [50]. In Lebanon the VHI sector boomed post 1992 due to gaps in public health insurance after the end of a 17 year civil that had left the public sector with very limited capacity and with a fragmented health system and health financing structure [51, 52]. Despite improvements in the government health sector, private sector provision has still remained dominant and patients faced high user charges. This may explain why VHI% increased substantially in the mid-2000s, coupled with a lack of effective control mechanisms, which led to rising premiums.

The decrease in VHI% in Morocco can be explained by an increase in GGHE% due to the expansion of compulsory public health insurance from the mid-2000s onwards, although the speed of decline may be slower than had been expected [53], also given the continued opting-out option for employers to provide VHI to their employees [54]. A similar explanation may be valid for Tunisia, where VHI play a supplementary but also primary (substitutive) role [55]. The lower VHI% rate found in Egypt can be explained by a lack of regulation conducive for the VHI sector to develop. This may explain why OOPs in the private sector did not turn into supplementary VHI [56].

In summary, the different trends found in this region can be related to diverse reasons, namely: limited growth in VHI% related to non-conducive conditions for the VHI market to expand (Egypt), or improvements in public provision and public health insurance (Tunisia, Morocco). Vice versa larger growth in VHI% was also due to gaps in public health insurance (Jordan, Lebanon).

#### European Region (EURO)

**Fig. 9:**
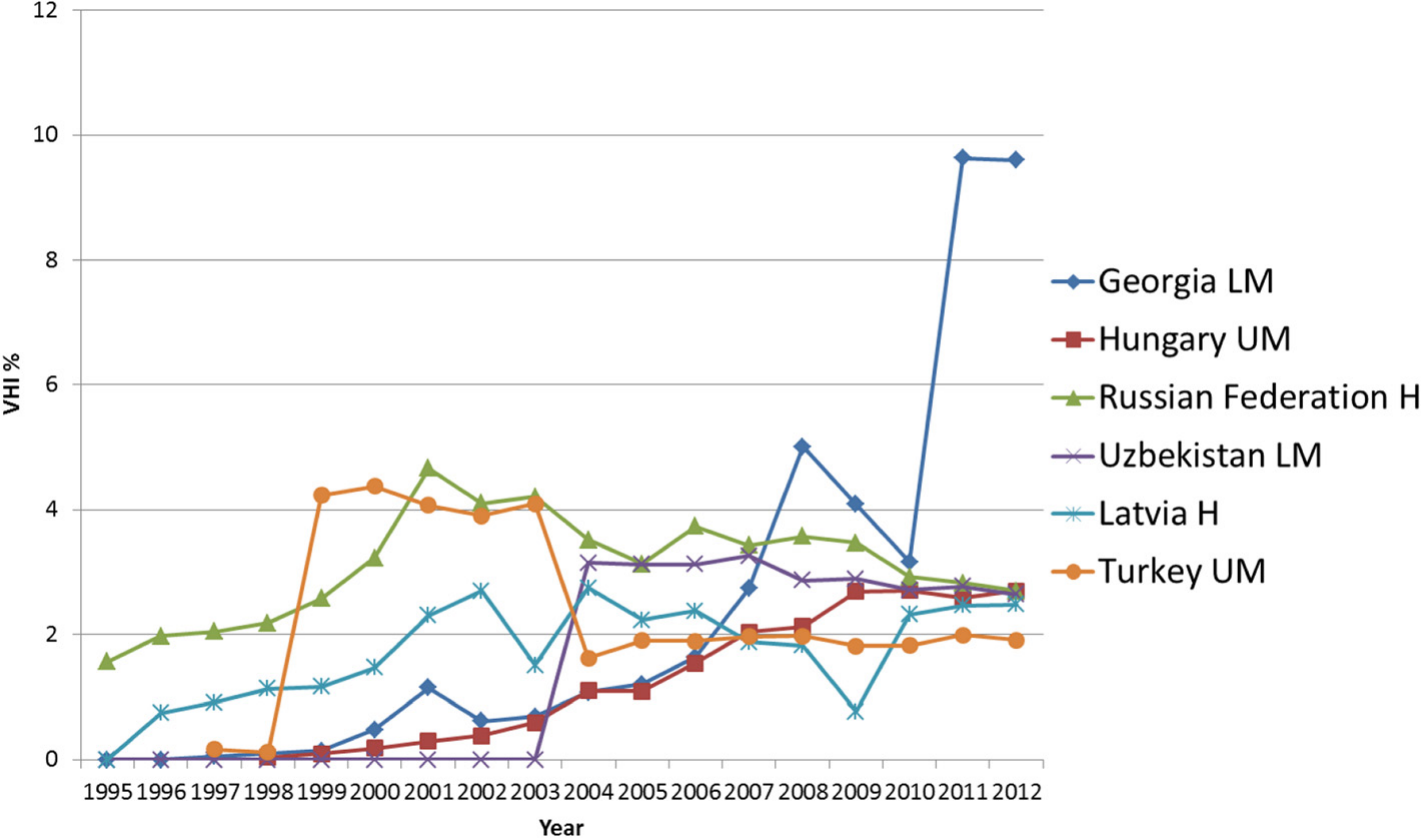
EURO Individual country trends of VHI%, 1995–2012

The availability of VHI expenditure data was very limited for the 23 EURO LMIC countries, with only 12 countries reporting VHI expenditure with less than five years of data missing. Out of these, six countries had a VHI% > 1 % in any year between 2008 and 2012 (Georgia, Hungary, Russian Federation, Uzbekistan, Latvia and Turkey). Based on the available data, EURO LMIC show the overall lowest average VHI%, only since 2011 is the average VHI% slightly lower in SEARO LMICs (see Fig. 3). It should be noted that the Russian Federation and Latvia (and Lithuania which did not have a VHI% >1 % between 2008 and 2012, so not included in the trend analysis) became HIC in 2012. All countries included with a VHI% > 1 % (see Fig. 9 for the detailed country analysis) have seen an overall rise in VHI% since 1995, but other than Hungary and Georgia, the VHI% decreases from the early 2000s onwards. Except for Georgia, VHI% remained below 5 % in all countries.

In Hungary, Russian Federation and Latvia, the GGHE% decreases, with both OOP% and VHI% increasing, which we argue is not a desirable way of equitably moving towards UHC. In contrast, in Georgia, Uzbekistan and Turkey, GGHE% and VHI% increases, while OOP% goes down (see Additional file 2). Some OOP expenditure is likely to have been turned into VHI, and as total private health expenditure is going down, the trend of these three countries is going in a more desirable direction towards UHC. However in terms of equity it may be preferable for a greater part of OOP% to be redirected into GGHE% rather than to VHI%.

More than half of the EURO countries assessed here have a hybrid financing system of payroll taxes and government budget transfers. With the exception of Georgia, the other remaining countries have a national health service in place. The legacy of the Soviet health system model, with its strong focus on central government funding and the absence of a private sector, would explain why the majority of ex-Soviet countries have very low VHI% (below 1 % or reported as 0 %). In the post-Soviet transition lack of trust in private institutions and affordability issues further contributed to this [11]. In the Russian Federation, Latvia and Hungary, which have a mandatory health insurance system that is also financed through budget revenues, VHI plays largely a complementary and/or supplementary role [7, 57, 58]. This is also the case in Uzbekistan with a state-run health system [6]. Of note the cost of VHI policies is not regulated and these are relatively high due to small risk pools and limited number of sales, which makes them predominately accessible only to higher income groups [59].

In Turkey, despite its growth, supplementary VHI plays a minor role so far. It covers solely one percent of the population, in particular higher income households that seek supplementary coverage. A major reason for the decline in VHI% in 2004 is linked to the Health Transformation Program starting 2003 with increased GGHE. This resulted in a decline of the share of the population covered by VHI [60].

In Georgia in 1995, the government introduced public health insurance. However after the Rose Revolution in 2003 the changing political ideology led to the abolishment of the public health insurance and VHI has become the principal mechanism for pre-payment of health services. Those not belonging to the defined groups of the poor and pensioners benefiting from subsidized health insurance had to revert to VHI as primary (principal) coverage. Accompanied by relatively weak regulation, this is reflected in the rise in VHI% from 2003 onwards [61].

In summary, VHI has largely a supplementary/complementary role in most EURO countries studied here, whereby higher income groups in particular demand VHI.

#### South-East Asian Region (SEARO)

VHI in its various roles has played a rather moderate role so far in SEARO, which is reflected in its very low average VHI% trend lines (see Fig. 3) compared to other regions. From 2011 it had the lowest VHI% average of all the regions.

**Fig. 10:**
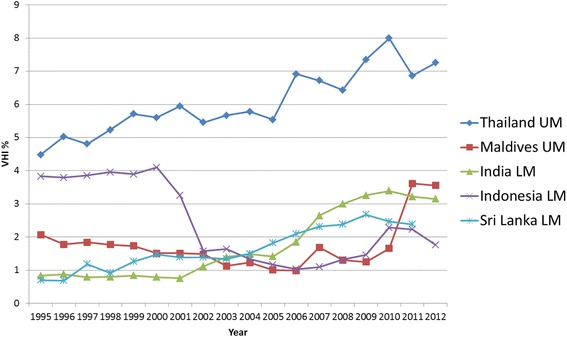
SEARO Individual country trends of PHI%, 1995–2012

Out of 10 SEARO LMIC only five (Thailand, Maldives, India, Indonesia and Sri Lanka) reported a VHI% > 1 % in any year between 2008 and 2012 (see Fig. 10). With the exception of Indonesia all countries have reported arise in VHI% since 1995. In two countries (Sri Lanka and Maldives), GGHE% decreases, while both OOP% and VHI% increase which is a less desirable scenario. In the remaining countries GGHE% increases and OOP% goes down, while VHI% goes up (in Thailand and India) or down (in Indonesia) (see Additional file 2).

Despite the moderate role of VHI% in SEARO, VHI expenditure per capita is growing gradually. One significant explanatory factor in SEARO is economic growth of parts of the region and the growing middle class with expanding health care demands. For example, for Thailand, Drechsler and Jütting reported that the growing middle classes are assumed to be the most likely consumers of supplementary VHI [9]. By contrast, in Indonesia limited VHI growth of approximately 20 ‘bapels’, which are HMO type organisations accessed through a voluntary insurance scheme, has been attributed to the belief that they are of poor quality and provide limited choice [62]. Additionally, coverage for formal sector workers was expanded and the government provided funding to subsidize coverage for poor population groups, called Jamkesmas [63], which led to an expansion of GGHE.

While India has one of the largest private provider health sectors in the world, VHI% is still relatively small (0.8 % in 1995–3.1 % in 2012), against a notably high share of OOP expenditure. The growth of India's VHI market is due to growing demands of an emergent wealthier middle class for supplementary/complementary VHI and the expansion of numerous CBHI schemes for the poorer populations [64]. During the 2000s, unclear guidance from India's regulatory bodies for insurers has been attributed as a factor for restricted growth [65].

In summary, economic growth and the growing middle-class with expanding health care demands can be seen as a common reason for a rising VHI% trend in particular in some countries in the SEARO region.

#### Western Pacific Region (WPRO)

As for SEARO countries, VHI has also played a rather moderate role so far in WPRO countries, which is reflected in the relatively low average VHI% trend lines for the region (see Fig. 3).

**Fig. 11:**
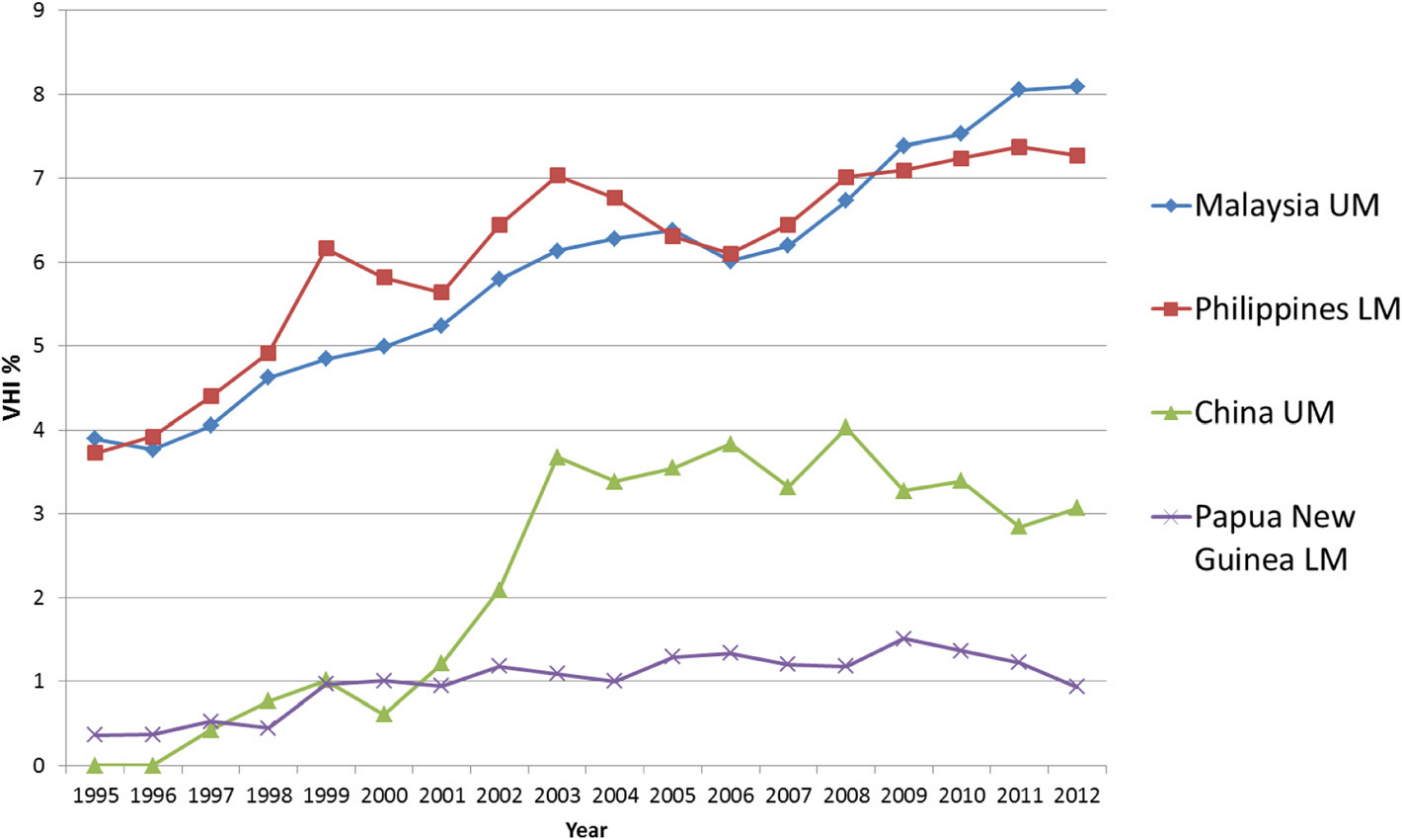
WPRO Individual country trends of VHI%, 1995–2012, non-pacific island states

**Fig. 12:**
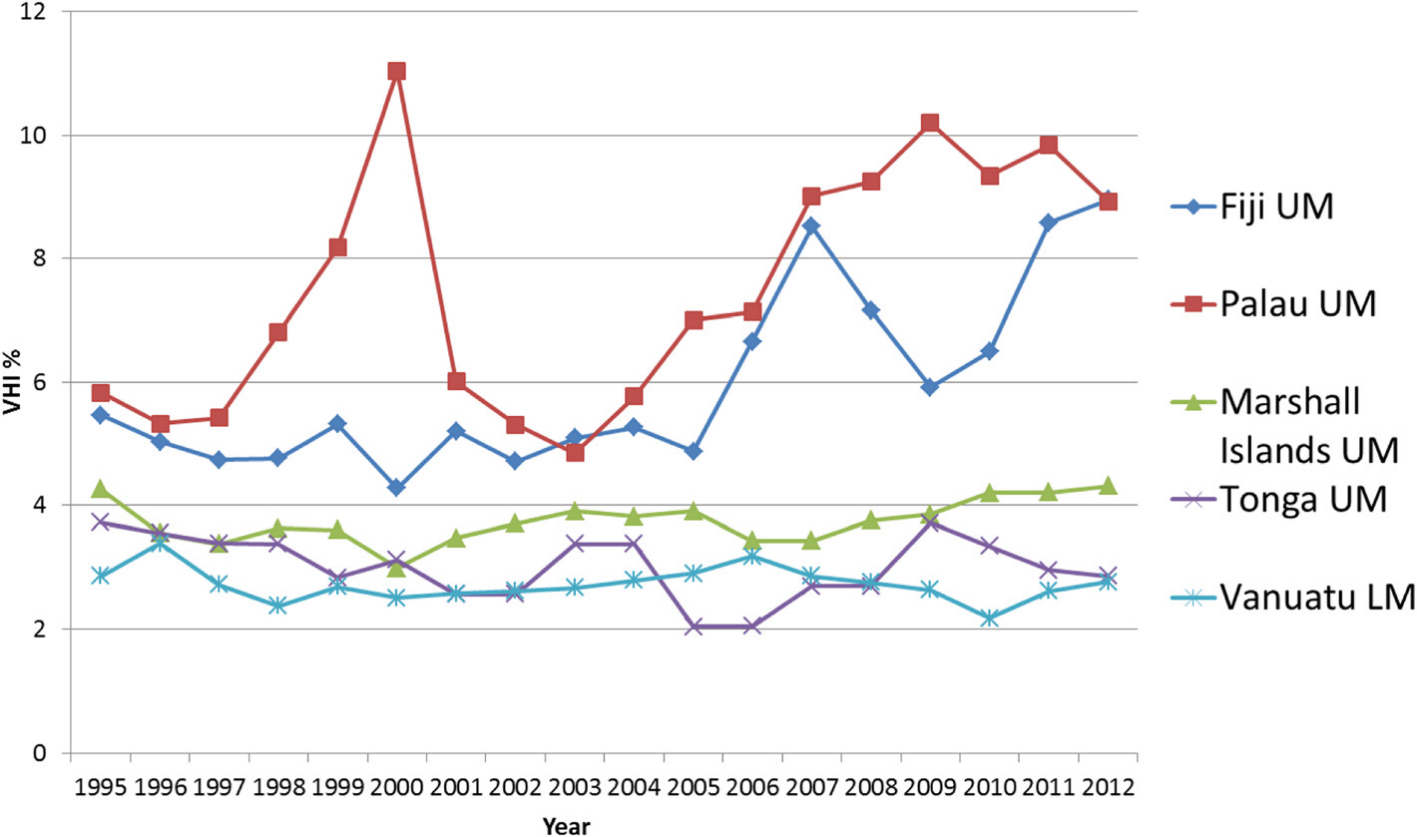
WPRO Individual country trends of VHI%, 1995–2012, pacific island states

There are 21 LMIC countries in WPRO (out of the 37 countries and areas that make up the region), with only one country not reporting on VHI expenditure. Nine of these reported a VHI% > 1 % in any one year between 2008 and 2012, five of which were pacific islands. Meanwhile ten countries reported that none of their THE was spent on VHI, the highest number across all the regions. Of the countries included in the country trend analysis, a general upwards trend can be seen in half of the countries (see Figs. 11 and 12). The data has been split into two graphs; non-pacific island states (Fig. 11) and pacific island states (Fig. 12)

.

GGHE% increases in five out of nine countries in WPRO, but there is no observable pattern with respect to changes in OOP% and VHI%. The remaining four countries where GGHE% decreases, both OOP% and VHI% increase (see Additional file 2); this is the less desirable scenario on the path of moving equitably towards UHC.

As for SEARO, one significant explanatory factor in WPRO for changes in VHI seems again to be economic growth of parts of the region and sectors of the population with expanding health care demands. In Malaysia, VHI mostly targets the more affluent parts of the population [66, 67], which together with increasing wealth in the upper middle classes has led to a gradual rise in VHI%. Similarly in the Philippines, even though its government has made efforts to expand the public health insurance package (PhilHealth), VHI% increased from 3.7 to 7.3 % between 1995 and 2012. This could be explained by an increase in additional services offered by VHI that are particularly attractive for the increasing middle class, as the PhilHealth package is limited in benefits [68].

For Vietnam, no VHI data is reported in the GHED. However, evidence and previous analysis of VHI trends in the country [11] reveals that despite substantial UHC extension efforts and government subsidies for the public health insurance system, duplicative VHI plays an important role [69]. A major problem is that VHI regulation is not coordinated with the ministry of health’s efforts to expand coverage via the Vietnam Social Security scheme. In China, the VHI sector was opened up to foreign companies in 1992 [70]. The modest increase in VHI% is largely the result of the expansion of the upper middle class that buys complementary/supplementary VHI coverage on top of the existing public health insurance programs [71].

Up to now, overall, VHI has played a rather marginal role in South East Asia and Western Pacific Regions in contrast to the economic potential of parts of the regions.

#### Global VHI% trends in LMIC

While data is missing for around one fifth of all LMIC over the period between 1995 and 2012, a slight upward trend of VHI% can be seen on average. VHI% increased in 68 LMIC (including the recent HICs). Yet the rise in regional averages of VHI% has been less than one percentage point (and 2 percentage points in EURO) over this whole period, and thus rather modest. Nonetheless, many individual AMRO and AFRO countries show larger increases. In a few countries the increase in VHI% between 1995 and 2012 was above 5 percentage points, namely in Botswana, Georgia, Suriname and Senegal. On the other hand, in 30 LMIC, VHI% decreased. While more recent VHI market developments largely relate to the expansion of supplementary/complementary coverage in many countries, in some countries with historic and markedly higher VHI% this has been driven by primary VHI. Existence and changes in duplicate coverage appear to have been less pertinent, although its relevance could only be established on the basis of a detailed country by country analysis of the actual VHI policy and national SHI laws.

It is also interesting to note the shifts between VHI%, OOP% and GGHE% between 1995 and 2012 to reveal how countries progress towards UHC: Out of the 74 countries included in the detailed individual country trend analyses (with VHI% > 1 % in any one year between 2008 and 2012), 29 countries had a rise in VHI% and GGHE%, while OOP% declined, but eight out of these still had OOP% > 40 %. Yet, seventeen countries had a rise in VHI% plus a rise in OOP% and a fall in GGHE%, which is an undesirable trend on the path towards UHC. Moreover, eight out of these 17 countries still had OOP% >40 % of THE (see Additional file 2).

## Discussion

From 193 countries worldwide in 2012, 163 had available data. Out of 138 LMIC, VHI expenditure was insignificant in 49 LMIC, i.e. with VHI% below 1 % or reported as 0 %. VHI% ranged from 1 % to 5 % in 56 countries of all income levels, with 39 of these being LMIC and 3 having recently become HIC. VHI% was above 5 % in 46 countries worldwide, 23 of these being LMIC and 3 being new HIC. In 2001, in comparison, VHI% was above 5 % in 39 countries worldwide, with 25 of these being LMIC then [8]. The absolute number of countries of all incomes with VHI% >5 % has increased between 1995 and 2012, from 36 to 46. The overall average global trend of VHI% in LMIC between 1995 and 2012 is slightly upwards. This increase has been noted by studies of NHA data prior to 2002 [10] and has also taken place over the last 10 years as observed in this study. Moreover, we need to keep in mind that underreporting and general difficulties in obtaining data may imply that VHI expenditure is actually higher than suggested by the data presented here.

Analysis of changes in OOP% between 1995 and 2012 indicates that increases in VHI% cannot be consistently linked with OOP% falling or being redirected into voluntary prepayment. This suggests that generally VHI is not effective in covering gaps in publicly financed coverage. There were also several countries where despite a rise in VHI% there has been a rise in OOP% coupled with a fall in GGHE%.

Diverse reasons for the VHI expenditure trends were identified in the previous section, from these three overarching themes emerge: 1) external influences; 2) government policies on the role of VHI and its regulation; and 3) willingness and affordability of various population segments to pay to enrol in VHI schemes. These themes are interrelated, and can increase or decrease VHI expenditure depending on the country context.

### External influences

External influences were one driving factor for increases in VHI expenditure. Several Latin American countries underwent structural adjustment programmes in the 1980s. International trade agreements to attract foreign investment, like in Georgia, and market oriented liberalization efforts have tended to encourage the growth of the private sector and the reduction of investment in the public sector. This attracted interest from overseas VHI companies and resulted in AMRO having the greatest proportion of LMICs with a VHI% >10 %. Likewise, in post-apartheid South Africa such policies led to the further growth of the private sector and overseas investment.

Another cause for VHI changes are development partners and international donors that influence health policy or provide external funding. They contributed significantly to the promotion of CBHI in terms of numbers of schemes, primarily in sub-Saharan Africa. However, the absolute VHI expenditure increases due to CBHI have been marginal, largely due to relatively low enrolment rates [22].

### Government policy on the role of VHI and its regulation

In most LMIC, VHI has so far played a limited role, whereas in some countries it has played a more important role. Yet, the role of VHI is often not adequately reflected in health sector policies and is largely absent in health financing policies or health financing strategies, as was revealed when screening such documents, which are available on the IHP+ platform [18]. In some strategies VHI is mentioned as complementing and supplementing health financing, but in what form, and what regulation will be required is often not specified.

One challenge is that VHI often does not fall under the policy and regulation of the ministry of health. When regulated by the Ministry of Finance or another ministry, their policy objective is often private sector growth (including VHI). If not aligned and regulated, this could contradict UHC and financial protection objectives.

The country analyses reveal that regulation has been weak overall, and this has had various effects. On the one hand, a general lack of regulation enhanced the expansion of primary as well as supplementary and complementary VHI in several countries. This has been documented for South Africa during apartheid and many parts of Latin America, where VHI was described to have developed in a “legal vacuum” coupled with laissez faire market oriented policies. VHI has also been noted to grow in post-conflict countries, such as Lebanon, potentially due to limited capacity of public institutions to regulate private industries. On the other hand, the lack of a regulatory framework such as in Mexico or India may have inhibited the anticipated development of primary (substitutive) or supplementary/complementary VHI. The absence of a secure legal framework for VHI companies to grow and profit can be a significant disincentive to investment.

This also seems to explain expenditure trends in the two large emerging economies China and India. These can be considered together as there has been speculation that India's and China's VHI market would expand rapidly, with their anticipated economic growth [9]. These countries have had the greatest % increases in VHI expenditure per capita in their respective region, but it is still within the average range. However trend analysis of overall VHI% growth for India since 1995 is still modest, and VHI% actually decreased in China since 2008. This perhaps less than anticipated VHI% growth has been attributed to inadequate clarity regarding government and health regulators’ policies on the role of VHI [65]. It should be emphasized however that aggregate figures for large countries with marked social gradients, such as India and China, may mask substantial increases in VHI expenditure within certain segments of the population. It should also be noted that VHI% are affected by changes in OOP and GGHE, which also contribute to THE.

Although less frequent, explicit government policy to expand VHI has played a role in some countries. For example, the promotion of CBHI as a key pillar for UHC expansion foremost in West African countries, the introduction of a regulatory framework and a federation of CBHIs to support their growth in Senegal, the promotion of principal VHI coverage in Georgia, or legislation to allow people to opt out of public health insurance in favour of substitutive VHI in Chile. In fact, although CBHI expenditure is rather marginal in Africa and Asia, its existence and promotion through donors and development partners has made it in some countries turn into a strong political actor and potentially an overvalued health financing mechanism. A crucial yet unrealistic role of CBHI contributing towards UHC has been ascribed by envisaging to cover the informal sector population which is often more than 80 % of the population. Assigning such a role to CBHI may have also potentially detracted governments in the past from expanding publicly financed coverage and from increasing GGHE.

In contrast explicit government regulation intended to limit VHI activity and increase investment in GGHE was seen in post-apartheid South Africa and post-military rule Chile, where social movements to diminish inequities precipitated a shift in government policies. However the success of such policies was limited by existing VHI industry interests operating in a primary VHI market. This is an important lesson for countries on the path to UHC, suggesting that although VHI may be considered to help increase pre-payment mechanisms and reduce financial risk in the first instance if vested interests become significant this may hinder future efforts to expand GGHE.

### Willingness and affordability to enrol in VHI schemes

A common issue across all regions is the emergence of a middle class with disposable income and the desire to access better quality health services. This is often the case when the services provided by the public system do not meet expectations, and in some countries has been catalysed by the labour unions of private companies negotiating VHI for their workers. The growth in purchasing power by a segment of the population is seen in emerging economies such as China, India and Thailand, and was or continues to be a cause of growth in VHI in its various roles in a diverse range of countries, such as several upper-middle income countries in Latin America, EURO countries as well as Jordan and Botswana. Of note existing inequality, as expressed by a high Gini-coefficient, was found to be correlated with a higher VHI% in previous studies [11].

On the other hand, various countries have experienced the consequences of excessive fragmentation and limited pooling of VHI, including CBHI schemes, which led to unaffordable rising premiums and/or unsustainable schemes, such as in Turkey, South Africa, Chile and Mexico. In some cases this was precipitated by economic crises.

#### Limitations

Important limitations around the quality of VHI data have been discussed in the methodology section. Unexplained sharp rises and falls of VHI expenditure which were seen in a few countries point to possible variations in the quality of data reporting. Country health financing system reviews revealed that VHI plays an important role in some countries, even when no VHI expenditure is recorded in the GHED or VHI is recorded as zero. This suggests that there may be considerable under-reporting from some countries, which confirms Drechsler and Jütting’s (2010) assessment [11]. For example, detailed health financing system reviews of Lesotho revealed that there is an important VHI market [72, 73] despite 0 % being recorded in GHED, as is the case for Nigeria [74, 75] and Vietnam [69]. Similarly, in Cameroon, there is no VHI expenditure reported despite a growing number of CBHI schemes in place covering about 2 % of the population [76]. It is also likely that VHI continues to play a role in Zimbabwe although unrecorded.

Underreporting is also due to the fact that GHED records VHI expenditure, rather than VHI premiums collected, i.e. revenues. In many countries, there are large profit margins in the VHI market, and thus household spending on VHI may be considerably above the reported VHI expenditure of financing agents [77].

The literature analysis also had its limitations. It identified sometimes patchy and at times contradictory causes to explain potential rises and falls in VHI% and VHI expenditure in individual countries. However, clear themes did emerge regarding potentially causal factors for changes in VHI. These are applicable taking into account different country contexts.

## Conclusion

Over the years several countries have paid insufficient attention to the role of VHI. Much of the initial expansion of VHI in many countries was a result of limited investment in the public sector as well as limited regulation and thereafter its maintenance due to vested interests, rather than a coordinated effort for it to play an ancillary role in achieving UHC. In some countries, VHI may have hindered moving equitably towards UHC. Current health financing strategies still put insufficient attention on specifying the role of VHI on the path to UHC. A systematic review of available health sector strategies would be useful to provide more evidence on existing gaps. It is of high importance that health financing strategies are clear about the role they give to VHI in their path towards UHC if previous oversights are not to be repeated.

Future research could explore in more depth possible associations between the rate of change in VHI expenditure and OOP, GGHE, THE or GDP as relative percentage changes or as per capita expenditure. Likewise more work is needed to disaggregate data to understand how these changes play out across different segments of the population. Furthermore, attention needs to be placed on improving data collection of VHI expenditure and its different roles at country level in order to better monitor country and global trends.

An increasing number of LMICs are committed to accelerating progress towards UHC and actively engage in reforming their health financing system. The focus needs to be on increasing GGHE in order to more equitably progress towards UHC. Likewise, there is a need to align policy objectives around private sector promotion (including VHI promotion) by the Ministry of Finance or Ministry of Industry with those of the Ministry of Health. It is clear that better-off population groups will often demand access to higher quality services from the private sector if the public sector does not provide these.

Regulation is also important in countries with still relatively slow growth in VHI expenditure, as this might change with an expanding middle class and economic growth. The point is thus to foster and regulate VHI in such a way that it contributes to equitable progress towards UHC. This is more likely to be so with VHI in its complementary or supplementary role, in comparison with primary and in particular substitutive VHI, with an opt-out mechanism from public health insurance, as well as duplicative coverage which can easily result in fragmentation and segmentation, weakening risk sharing, and reduced solidarity. In conclusion, there is a strong need to wisely manage VHI in order to contribute to a country’s endeavour to progress equitably towards UHC.

## Additional files

Additional file 1: LMIC excluded from individual country trend analysis 1995–2012. Presents countries not further considered in the analysis (DOCX 19 kb). Additional file 2: Countries’ rise or fall in GGHE%, VHI% and OOP% between 1995–2012. Presents for each country, organized by regions, the direction of change in OOP and VHI as a % of THE set against a rise or fall in GGHE as a % of THE, as well as grouping of countries into three levels of OOP% in 2012. (DOCX 19 kb).

## Abbreviations

AFRO: African Region
AMRO: Region of the Americas
AUGE: Plan de Acceso Universal con Garantias Explicitas (Chile)
CBHI: Community-based health insurance
CNAM: Caisse National d’Assurance Maladie (Tunisia)
EMRO: East Mediterranean Region
EURO: European Region
GDP: Gross domestic product
GGHE: General government health expenditure
GGHE%: General government health expenditure as a percentage of total health expenditure
GHED: Global Health Expenditure Database
HIC: High-income countries
HMO: Health Maintenance Organisation
LMIC: Low- and middle-income countries
NHA: National Health Accounts
OECD: Organisation for Economic Co-operation and Development
OOP: Out-of-pocket payments
OOP%: Out-of-pocket expenditure as a percentage of total health expenditure
VHI: Voluntary health insurance
VHI%: Voluntary health insurance expenditure as a percentage of total health expenditure
PvtHE: Private health expenditure
SEARO: South East Asian Region
SHA: System of Health Accounts
THE: Total health expenditure
UHC: Universal health coverage
WHO: World Health Organization
WPRO: Western Pacific Region

## References

[1] WHO (2010). The World Health Report: Health Systems Financing - The path to universal health coverage.

[2] WHO (2014). Making fair choices on the path to universal health coverage: final report of the WHO consultative group on equity and universal health coverage.

[3] Mossialos E, Thomson SM: Voluntary health insurance in the European Union. Funding health care: options for Europe 2004:128.

[4] Colombo F, Tapay N: Private health insurance in OECD countries: the benefits and costs for individuals and health systems. Towards High-Performing Health Systems: OECD working paper No 15. DELSA/ELSA/WD/HEA(2004)6.

[5] OECD (2004). The OECD Health Project - Private Health Insurance in OECD Countries.

[6] Thomson S, Mossialos E: Private health insurance in the European Union. European Commission; 2009

[7] Thomson S: Chapter 11: What role for voluntary health insurance? In Implementing health financing reform. Edited by Kutzin J, Cashin C, Jakab M: World Health Organisation on behalf of the European Observatory on Health Systems and Policies. Copenhagen: WHO Regional Office for Europe: 2010.

[8] Sekhri N, Savedoff W (2005). Private health insurance: implications for developing countries. Bull World Health Organ.

[9] Drechsler D, Jutting J (2005). Private Health Insurance in Low and Middle Income Countries.

[10] Drechsler D, Jutting J (2007). Different countries, different needs: The role of private health insurance in developing countries. J Health Polit Policy Law.

[11] Drechsler D, Jütting J, Preker AS, Zweifel P, Schellekens OP (2010). Six Regions, One Story. Global marketplace for private health insurance: strength in numbers.

[12] Thomson S: What role for voluntary health insurance? Cautionary tales from Europe. In WHO Advanced Course on Health Financing for Universal Health Coverage. Barcelona: WHO; 2015.

[13] Global Health Expenditure Database [http://apps.who.int/nha/databasets]. Accessed date 1 Dec 2014.

[14] OECD, Eurostat, WHO: A System of Health Accounts: 2011 Edition. OECD Publishing; 2011.

[15] Siskou O, Kaitelidou D, Economou C, Kostagiolas P, Liaropoulos L (2009). Private expenditure and the role of private health insurance in Greece: status quo and future trends. Eur J Health Econ.

[16] WHO: Guide to producing national health accounts: with special applications for low-income and middle-income countries. Geneva: World Health Organization; 2003.

[17] OECD (2000). A System of Health Accounts Version 1.0.

[18] Country Planning Cycle Database [http://www.internationalhealthpartnership.net/en/tools/country-planning-database/]. Accessed date 16 Feb 2015.

[19] Ndiaye P, Soors W, Criel B (2007). Editorial: A view from beneath: Community health insurance in africa. Tropical Med Int Health.

[20] Musango L, Makaka A, Muhongerwa D, Kalisa I, Elovainio R (2013). Strategies towards universal health coverage in Rwanda: Lessons learned from extending coverage through mutual health organizations.

[21] Twahirwa A (2008). Sharing the burden of sickness: mutual health insurance in Rwanda. Bull World Health Organ.

[22] Acharya A, Vellakkal S, Taylor F, Masset E, Satija A, Burke M, Ebrahim S: The impact of health insurance schemes for the informal sector in low-and middle-income countries: a systematic review. The World Bank Research Observer 2012:lks009.

[23] Krämer A, Haupt S, Coetzer v, von Blomberg I: Health and Medicines Sector Market Assessment in Botswana, Lesotho, Namibia and South Africa. (development E-Esf ed.; 2014.

[24] Gilson L, McIntyre D, Evans T, Whitehead M, Diderichsen F, Bhuiya A, Wirth M (2001). South Africa: addressing the legacy apartheid. Challenging Inequities in Health: From Ethics to Action: From Ethics to Action.

[25] Shisana O, Rehle T, Louw J, Zungu-Dirwayi N, Dana P, Rispel L (2006). Public perceptions on national health insurance: Moving towards universal health coverage in South Africa. S. Afr. Med. J.

[26] Fish T, Ramjee S (2007). Unaffordable medical scheme contributions: A barrier to access to private health cover in South Africa. Afr J Bus Manage.

[27] McLeod H, Grobler P (2010). Risk equalisation and voluntary health insurance The South Africa experience. Health Policy.

[28] Global Health Expenditure Database 1995-2012 [http://apps.who.int/nha/database/Select/Indicators/en]. Accessed date 1 Dec 2014.

[29] Baker PA (2010). From apartheid to neoliberalism: Health equity in post-apartheid South Africa. Int J Health Serv.

[30] Mooney GH, McIntyre DE (2008). South Africa: a 21st century apartheid in health and health care?. Med J Aust.

[31] Musango L, Nabyonga J, Elovainio R, Cheruiyot S (2013). State of health financing in the African Region.

[32] PAHO: Perfil de sistemas y servicios de salud, Suriname. First edition; 2002.

[33] Hindori M: White Paper - Health Sector Reform in Suriname. Paramaribo: Ministry of Health & Inter-American Development Bank; 2002.

[34] Alvarez L, Salmon J, Swartzman D (2011). The Colombian health insurance system and its effect on access to health care. Int J Health Serv.

[35] Rathe M, Moline A (2011). The health system of Dominican Republic. Salud Publica De Mexico.

[36] Laver R: Private health care in Latin America: emerging opportunities. La Jolla (CA): Institute of the Americas 2000:1–12.

[37] Knaul FM, González-Pier E, Gómez-Dantés O, García-Junco D, Arreola-Ornelas H, Barraza-Lloréns M, Sandoval R, Caballero F, Hernández-Avila M, Juan M (2012). The quest for universal health coverage: achieving social protection for all in Mexico. Lancet.

[38] Stocker K, Waitzkin H, Iriart C (1999). The exportation of managed care to Latin America. N Engl J Med.

[39] Affat RP: Brazilian Private Health Care Market—Ready for Liftoff. In Article from: International News, vol. Issue No.55: Society of Actuaries; 2012.

[40] Marten R, McIntyre D, Travassos C, Shishkin S, Longde W, Reddy S, Vega J (2014). An assessment of progress towards universal health coverage in Brazil, Russia, India, China, and South Africa (BRICS). Lancet.

[41] Belmartino LS, Bloch C: El sector salud en Argentina: actores, conflictos de intereses y modelos organizativos, 1960–1985; Health sector in Argentina: actors, conflicts of interest and organizing models 1960–1985. Argentina: Organización Panamericana de la Salud; 1994.

[42] Csillag C (1998). Brazil rules on private health plans. Lancet.

[43] Lloyd-Sherlock P (2005). Health sector reform in Argentina: a cautionary tale. Soc Sci Med.

[44] Cotlear D, Gómez-Dantés O, Knaul F, Atun R, Barreto IC, Cetrángolo O, Cueto M, Francke P, Frenz P, Guerrero R: Overcoming social segregation in health care in Latin America. The Lancet. 2014;385(9974):1248–259. 10.1016/S0140-6736(14)61647-0 25458715

[45] Atun R, de Andrade LOM, Almeida G, Cotlear D, Dmytraczenko T, Frenz P, Garcia P, Gómez-Dantés O, Knaul FM, Muntaner C: Health-system reform and universal health coverage in Latin America. The Lancet 2014;385(9974):1230–247. 10.1016/S0140-6736(14)61646-9 25458725

[46] Sanchez M (2005). Migracion de Afiliados en el Sistema Isapre. Working paper.

[47] Cid C, Torche A, Herrera C, Bastías G, Barrios X, Públicas CdP: Bases para una reforma necesaria al seguro social de salud chileno. Centro de Políticas Públicas Propuestas para Chile Santiago: Universidad Católica de Chile 2013:183–219.

[48] Al-Qudah HSS. Hand in Hand with Jordanian Health Care Insurance: A Challenge of Improvements. Int J Bus Soc Sci. 2011;2(3):111-21.

[49] WHO: Health Systems Profile - Jordan. Regional Health Systems Observatory EMRO; 2006.

[50] Preker AS, Scheffler RM, Bassett MC. Private voluntary health insurance in development: friend or foe?: Washington DC: World Bank Publications; 2007.

[51] Kronfol NM (2006). Rebuilding of the Lebanese health care system: health sector reforms. East Mediterr Health J.

[52] El-Kak F, Khawaja M, Salem M, Zurayk H (2009). Care-seeking behavior of women with reproductive health problems from low-income areas of Beirut. Int J Gynecol Obstet.

[53] El-Idrissi DZE, Miloud K, Belgacem S (2008). Constraints and obstacles to social health protection in the Maghreb: the cases of Algeria and Morocco. Bull World Health Organ.

[54] World Bank (2014). Pour une universalisation de la couverture medicale au Maroc. Note d'orientation strategique. Rapport technique.

[55] WHO: Analyse du système de financement de la santé en Tunisia. Technical Report. Tunis; 2013.

[56] Salah H (2007). Mapping of Healthcare Financing in Eastern Mediterranean Region.

[57] Popovich L, Potapchik E, Shishkin S, Richardson E, Vacroux A, Mathivet B: Health Systems in Transition. Russian Federation, Health system review, vol. 13; 2011. 22455875

[58] Chernichovsky D, Potapchik E (1997). Health system reform under the Russian health insurance legislation. Int J Health Plann Manag.

[59] Ahmedov M, Azimov R, Alimova V, Rechel B: Health Systems in Transition. Uzbekistan, Health system review. Health 2007, 9. 25689490

[60] Atun R, Aydın S, Chakraborty S, Sümer S, Aran M, Gürol I, Nazlıoğlu S, Özgülcü Ş, Aydoğan Ü, Ayar B (2013). Universal health coverage in Turkey: enhancement of equity. Lancet.

[61] Chanturidze T, Ugulava T, Durán A, Ensor T, Richardson E: Health Systems in Transition. Georgia, Health system review. World Health Organization 2009, on behalf of the European Observatory on Health Systems and Policies. Copenhagen: WHO Regional Office for Europe. 2009;11(8).

[62] Ramesh M, Wu X (2008). Realigning public and private health care in southeast Asia. Pacific Review.

[63] Aji B, De Allegri M, Souares A, Sauerborn R (2013). The Impact of Health Insurance Programs on Out-of-Pocket Expenditures in Indonesia: An Increase or a Decrease?. Int J Environ Res Public Health.

[64] Michielsen J, Criel B, Devadasan N, Soors W, Wouters E, Meulemans H (2011). Can health insurance improve access to quality care for the Indian poor?. Int J Qual Health Care.

[65] Battacharjya AS, Sapra PK (2008). Health insurance in China and India: Segmented roles for public and private financing. Health Aff.

[66] Yu CP, Whynes DK, Sach TH (2008). Equity in health care financing: The case of Malaysia. Int J Equity Health.

[67] Chua HT, Cheah JC (2012). Financing Universal Coverage in Malaysia: a case study. BMC Public Health.

[68] DOH, Philhealth: Health care financing strategy of the Philippines 2010–2020. The path towards a social health insurance model Working Paper. Manila: DOH and Philhealth 2009.

[69] Van Tien T, Thi Phuong H, Mathauer I, Thi Kim Phuong N: A health financing review of Vietnam with a focus on social health insurance. Ha Noi, Vietnam: World Health Organization; 2011.

[70] Bhat R, Babu SK: Health insurance and third party administrators: Issues and challenges. Economic and Political Weekly 2004:3149–3159.

[71] Liu H, Gao S, Rizzo JA (2011). The expansion of public health insurance and the demand for private health insurance in rural China. China Econ Rev.

[72] Mwase T, Kariisa E, Doherty J, Hoohlo-Khotle N, Kiwanuka-Mukiibi P, Williamson T: Lesotho Health Systems Assessment 2010. In Bethesda, MD: Health Systems vol. 20. pp. 20: Abt Associates Inc.; 2010:20.

[73] Mathauer I, Musango L, Carrin G, Mthethwa K (2007). Report of the technical support mission for the feasibility assessment and financial projection results for a Social Health Insurance Scheme in Lesotho: exploring possible options.

[74] Odeyemi I, Nixon J. Assessing equity in health care through the national health insurance schemes of Nigeria and Ghana: a review-based comparative analysis. Int J Equity Health. 2013;12(9):1-18. 10.1186/1475-9276-12-9 PMC3626627 23339606

[75] Ejughemre UJ (2014). Accelerated reforms in healthcare financing: The need to scale up private sector participation in Nigeria. Int J Health Policy Manag.

[76] Sikod F, Abba I (2011). Ciblage de pauvres dans le financement communautaire de sante au Cameroun.

[77] Mathauer I, Nicolle E (2011). A global overview of health insurance administrative costs: what are the reasons for variations found?. Health Policy.

[78] Preker AS, Zweifel P, Schellekens OP. Global marketplace for private Health Insurance: strength in numbers. Washington DC: World Bank Publications; 2010.

[79] Buchmueller TC, Couffinhal A (2004). Private health insurance in France.

[80] Thomson S. Chapter 11: What role for voluntary health insurance? In: Kutzin J, Cashin C, Jakab M. Implementing health financing reform, vol 7. World Health Organisation on behalf of the European Observatory on Health Systems and Policies. Copenhagen: WHO Regional Office for Europe; 2010.

[81] Mathauer I, Cavagnero E, Vivas G, Carrin G: Health financing challenges and institutional options to move towards universal coverage in Nicaragua. Background paper24 for the World Health Report: Health Systems Financing: the Path to Universal Coverage 2010.

