# Supplementary material for: Voluntary Health Insurance expenditure in low- and middle-income countries: Exploring trends during 1995–2012 and policy implications for progress towards universal health coverage
Source: Int J Equity Health. 2016 Apr 18;15:67. doi: 10.1186/s12939-016-0353-5 (PMC4836104; doi:10.1186/s12939-016-0353-5)
Supplement: Additional file 1: — LMIC excluded from individual country trend analysis 1995–2012. Presents countries not further considered in the analysis (DOCX 19 kb). [file 12939_2016_353_MOESM1_ESM.docx]

Additional file 1: LMIC excluded from individual country trend analysis 1995-2012

| **WHO Region** | **No VHI expenditure data recorded throughout 1995-2012** | **Missing ≥ 5 years of VHI data during 1995-2012** | **Zero VHI expenditure recorded throughout 1995-2012** | **Recorded VHI% ˂1% during the last 5 years of the observation period (2008-2012)** |
| --- | --- | --- | --- | --- |
| **AFRO** | Mozambique  Cameroon  Lesotho | Zimbabwe  Seychelles  South Sudan  Liberia | Comoros  Eritrea  Guinea-Bissau  Angola  Sao Tome and Principe | Cape Verde  Guinea  Sierra Leone  Ethiopia  Mali  Chad  Uganda  Burundi  Mauritania  Central African Republic |
| **AMRO** | Haiti  Guyana  Grenada  Saint Vincent and the Grenadines |  | Cuba | Saint Lucia |
| **EMRO** | Iraq  Syrian Arab Republic | Afghanistan  Somalia | Libyan Arab Jamahiriya | Yemen  Sudan  Djibouti  Pakistan |
| **EURO** | Kyrgyzstan  Turkmenistan  Montenegro  The former Yugoslav Republic of Macedonia | Tajikistan  Republic of Moldova  Armenia  Serbia  Bulgaria  Romania  Bosnia and Herzegovina | Albania | Ukraine  Lithuania  Azerbaijan  Belarus  Kazakhstan |
| **SEARO** |  |  | Myanmar  Timor-Leste | Nepal  Bangladesh  Bhutan |
| **WPRO** | Viet Nam |  | Cook Islands  Nauru  Niue  Cambodia  Kiribati  Micronesia (Federated States of)  Mongolia  Samoa  Solomon Islands  Tuvalu | Lao People's Democratic Republic |
