# Supplementary material for: Voluntary Health Insurance expenditure in low- and middle-income countries: Exploring trends during 1995–2012 and policy implications for progress towards universal health coverage
Source: Int J Equity Health. 2016 Apr 18;15:67. doi: 10.1186/s12939-016-0353-5 (PMC4836104; doi:10.1186/s12939-016-0353-5)
Supplement: Additional file 2: — Countries’ rise or fall in GGHE%, VHI% and OOP% between 1995–2012. Presents for each country, organized by regions, the direction of change in OOP and VHI as a % of THE set against a rise or fall in GGHE as a % of THE, as well as grouping of countries into three levels of OOP% in 2012. (DOCX 19 kb). [file 12939_2016_353_MOESM2_ESM.docx]

Additional file 2: Countries’ rise or fall in GGHE%, VHI% and OOP% between 1995-2012

|  | **OOP% ↑**  **VHI% ↑** | **OOP% ↑**  **VHI% ↓** | **OOP %↓**  **VHI% ↑** | **OOP% ↓**  **VHI% ↓** |
| --- | --- | --- | --- | --- |
| **AFRO** | | | | |
| GGHE%↓ | *Namibia*  **Kenya** | **Mauritius** |  | United Republic of Tanzania |
| GGHE%↑ |  | Ghana | Zambia  Madagascar  *South Africa*  Senegal  Rwanda  **Benin**  Burkina Faso  **Nigeria**  *Botswana*  **Cote d’Ivoire**  Democratic Republic of Congo  *Algeria*  Congo | *Swaziland*  *Malawi*  **Togo**  **Niger**  *Gambia*  **Gabon** |
| **AMRO** | | | | |
| GGHE%↓ | Costa Rica  Nicaragua  **St Kitts & Nevis**  **Honduras**  **Venezuela** | **Ecuador** | **Guatemala** |  |
| GGHE%↑ | *Uruguay*  *Suriname* |  | Jamaica  Brazil  *Colombia*  Panama  Belize  El Salvador  Dominica  **Mexico** | Chile  Dominican Republic  Argentina  Antigua & Barbuda  **Paraguay**  Bolivia  Peru |
| **EMRO** | | | | |
| GGHE%↓ | **Egypt** |  | **Iran** |  |
| GGHE%↑ | Jordan | **Morocco** | **Lebanon** | Tunisia |
| **EURO** | | | | |
| GGHE%↓ | Hungary  Russian Federation  Latvia |  |  |  |
| GGHE%↑ |  |  | **Georgia**  **Uzbekistan**  *Turkey* |  |
| **SEARO** | | | | |
| GGHE%↓ | **Maldives**  **Sri Lanka** |  |  |  |
| GGHE%↑ |  |  | *Thailand*  **India** | **Indonesia** |
| **WPRO non-pacific island states** | | | | |
| GGHE%↓ | **Philippines**  *Papua New Guinea* |  |  |  |
| GGHE%↑ | Malaysia |  | China |  |
| **WPRO pacific island states** | | | | |
| GGHE%↓ | Fiji  *Marshall Islands* |  |  |  |
| GGHE%↑ |  |  | *Palau* | *Tonga*  *Vanuatu* |

Legend:

*Italic: OOP% <20% of THE*, Underline: OOP% ≥ 20% ≤ 40% of THE, **Bold: OOP% > 40% of THE**

↑ **=** share has increased**;** ↓ **=** share has decreased over the period of 1995-2012
